# The experience of conducting collaborative and intensive pragmatic qualitative (CLIP-Q) research to support rapid public health and healthcare innovation

**DOI:** 10.3389/fsoc.2022.970333

**Published:** 2022-09-15

**Authors:** Jeremy Horwood, Christalla Pithara, Ava Lorenc, Joanna M. Kesten, Mairead Murphy, Andrew Turner, Michelle Farr, Jon Banks, Sabi Redwood, Helen Lambert, Jenny L. Donovan, NIHR ARC West Behavioural and Qualitative Science Team

**Affiliations:** ^1^The National Institute for Health and Care Research Applied Research Collaboration West (NIHR ARC West), University Hospitals Bristol and Weston NHS Foundation Trust, Bristol, United Kingdom; ^2^Population Health Sciences, Bristol Medical School, University of Bristol, Bristol, United Kingdom; ^3^The NIHR Health Protection Research Unit in Behavioural Science and Evaluation, University of Bristol, Bristol, United Kingdom; ^4^The South West Academic Health Science Network, Exeter, United Kingdom

**Keywords:** qualitative methods, rapid qualitative methods, rapid qualitative research, qualitative health research, rapid appraisal, applied health research

## Abstract

A key challenge for qualitative methods in applied health research is the fast pace that can characterize the public health and health and care service landscape, where there is a need for research informed by immediate pragmatic questions and relevant findings are required quickly to inform decision-making. The COVID-19 pandemic accelerated the pace at which evidence was needed to inform urgent public health and healthcare decision-making. This required qualitative researchers to step up to the challenge of conducting research at speed whilst maintaining rigor and ensuring the findings are credible. This article illustrates how working with multidisciplinary, collaborative teams and the tailoring of qualitative methods to be more pragmatic and efficient can provide timely and credible results. Using time-limited case studies of applied qualitative health research drawn from the work of the Behavioral and Qualitative Science Team from the National Institute for Health and Care Research Applied Research Collaboration West (NIHR ARC West), we illustrate our collaborative and intensive pragmatic qualitative (CLIP-Q) approach. CLIP-Q involves (i) collaboration at all stages of the design, conduct and implementation of projects and, where possible, co-production with people with lived experience, (ii) an intensive team-based approach to data collection and analysis at pace, and (iii) pragmatic study design and efficient strategies at each stage of the research process. The case studies include projects conducted pre COVID-19 and during the first wave of the pandemic, where urgent evidence was required in weeks rather than months to inform rapid public health and healthcare decision making.

## Introduction

Qualitative researchers working in public health and health service settings face challenges to meet the demands of short timescales where findings are needed to inform rapid decision-making (Bamberger and Mabry, 2019). The COVID-19 pandemic amplified the need for rapid findings. Rapid methods are not new ADDIN EN.CITE (Scrimshaw and Hurtado, 1987; Bentley et al., 1988; Manderson and Aaby, 1992) with Scrimshaw and Hurtado publishing an introduction to conducting rapid methods in 1987 (Scrimshaw and Hurtado, 1987). Beebe identified more than 20 approaches reported under a range of terms and labels (Beebe, 2001), but despite the range in terminology, these “rapid evaluation and assessment method” (REAM) approaches share similar features when it comes to their purpose, as well as the design, methods and techniques proposed (Beebe, 2001; Mcnall and Foster-Fishman, 2007). For a more detailed description and comparison between the main rapid approaches see Mcnall and Foster-Fishman (2007), Nunns (2009), Beebe (2014), Vindrola-Padros and Johnson (2020), and Vindrola-Padros et al. (2021). The various REAM approaches were developed, particularly in the field of anthropology and international health and development, to meet the demand for timely results in rapidly changing situations while balancing speed and trustworthiness (Beebe, 2001; Malcolm and Aggleton, 2004). Qualitative health researchers have drawn from REAM approaches to provide participants views in short timescales (Mcmullen et al., 2011; Charlesworth and Baines, 2015), with aims and design guided by pragmatic considerations (Beebe, 2001; Vindrola-Padros and Johnson, 2020).

As qualitative researchers working in one of England's fifteen National Institute for Health and Care Research Applied Research Collaborations (NIHR ARCs), we recognize the benefits and challenges involved in undertaking intensive qualitative research within collaborative, multi-stakeholder teams. ARCs are partnerships between academic institutions and health and care systems, designed to integrate academic research into health and care practice. We aim to contribute to current debates around the use of “rapid” qualitative methods in applied health research, by describing, with the aid of three NIHR ARC West case studies (Box 1), our collaborative and intensive pragmatic qualitative (CLIP-Q) approach to deliver urgent high-quality research.

Box 1 **ARC West case study collaborative and intensive pragmatic qualitative projects.**

**Low vs. high dead space syringes study: user preferences and attitudes study**

This study aimed to find out whether people who inject drugs (PWID) are willing to switch from using a high dead space, to using a low dead space syringe to inject drugs. High dead space syringes have been traditionally used by needle exchange services, however low dead space syringes have been found to be safer and to reduce the chance of spreading infections when re-used or shared between users. Interviews were conducted with PWID, and volunteers and professionals who work with them to explore preferences and attitudes to low dead space syringes. The study was a collaboration between NIHR ARC West, NIHR HPRU in Behavioral Science and Evaluation, Bristol City Council, Public Health England and Bristol Drugs Project, a provider of harm reduction services in Bristol. People who use the service were included in the project steering group to provide advice and guidance to the research team. Participation of PWID was extended to them co-creating knowledge alongside the researchers by helping in attributing meaning to findings, and co-producing harm reduction materials to implement research findings. To accelerate the pace and scale of the rollout and uptake of low dead space syringes, service users co-produced seven posters, a booklet and a series of short animations, refining the messages, language and designs following each round of feedback and helping with dissemination and plans for implementation. Findings from the research contributed to the NICE surveillance proposal consultation on Needle and Syringe programmes (NICE guideline PH52), and two academic papers have been published outlining the main findings and the co-production process (Kesten et al., 2017; Hussey et al., 2019).

**Rapid COVID-19 intelligence to improve primary care response (RAPCI) project**

The RAPCI project was a longitudinal investigation into how GP practices were coping during the first COVID- 19 lockdown, and how they dealt with the rapid implementation of remote consulting, challenges faced, and solutions developed. The study was a collaboration between NIHR ARC West, Bristol, North Somerset and South Gloucestershire Clinical Commissioning Group (BNSSG CCG) and One Care (a GP federation of 77 general practices across BNSSG). The study rapidly recruited 21 GP practices. There were 87 interviews conducted in four, 2-3 week, rounds between May to July 2020, with 41 practice staff participants. In addition, anonymised patient record data (n= 350,966 patients) from the 21 practices were analyzed to examine how the volume and type of consultations with patients change over the period April to July 2020, compared to the same period in 2019. Findings were rapidly fed back to BNSSG CCG at weekly COVID-19 Primary Care Cell meetings to help inform their pandemic response. We published 5 rapid reports, which varied from 4 to 20 pages and had bullet point-descriptions of findings and recommendations for easy access. The rapid reports were published online and disseminated nationally via social media and professional networks. Findings were included in reports to UK government's Scientific Advisory Group for Emergencies (SAGE), the Royal College of General Practitioners (RCGP) and NHS England. Findings were also presented to the Department of Health and Social Care (DHSC) to inform their COVID-19 response. The team published three journal papers (Murphy et al., 2021; Scott et al., 2021; Turner et al., 2021) with preprint versions posted online prior to formal peer review and disseminated via twitter.

**Back to School Study**

The study investigated student, parent/carer and secondary school staff attitudes toward school COVID-19 mitigation measures. The study was a collaboration between NIHR ARC West and Bristol City Council. Between July - September 2020 interviews were conducted with 17 secondary school pupils, 20 parents and 13 school staff to rapidly investigate views on managing COVID-19 infections in schools ahead of school campuses opening in September 2020. Results were rapidly fed back to local authorities, schools and national policy makers and 2 rapid reports published online. Findings were included in Public Health England Behavioral Science Cell literature reports and disseminated to schools across the region and the Bristol City and North Somerset Council's Multiagency Children's COVID-19 response groups. Findings were published in BMJ Pediatrics (Lorenc et al., 2021) and a pre-print of the paper was disseminated to SPI-B (the behavioral science subgroup to the UK government's Scientific Advisory Group for Emergencies (SAGE) and findings presented to the Department for Education and submitted to two parliamentary enquiries into the impact of COVID-19 on education.

## Approaches to collaborative and intensive pragmatic qualitative methods

The CLIP-Q approach can inform each stage of the research process, as summarized in Table 1 and explained below.

**Table 1:** Summary of CLIP-Q design considerations.

**Stage in research process**	**Key design considerations**
Project set up and management	• Appropriate funding allocation to support the rapid project and meaningful collaboration • Embed collaboration at all levels: • Organizational – with decision-makers • Professional – with practitioners • Community organizations • Public involvement with people with lived experience • Team working within the research team • Be clear about the terms of reference - outline and document roles, responsibilities, expectations and time commitment of individuals involved in the project at initial meetings. • Work with collaborators to rapidly understand the context and setting of the research. • Planning – have clear timeline and milestones but be mindful that rapid research can be exhausting for the researchers involved, so ensure workloads and deadlines are realistic and acceptable. • Discuss where the blockages may be at the start of the project and plan strategies to avoid them – for example identifying potential recruitment issues. • Identify key audiences for research findings and agree the best means of communication across organizations, doing the groundwork in identifying and informing key people and embedding dissemination activities at an early stage.
Establishing the study aims	• Identify with partners priority needs and the depth and scope of research • Be open and flexible to changing priorities if situation being evaluated rapidly evolves • Ensure the research question is focused on key critical issues • Be clear about the potential impact of the project – what we are working toward
Participant sampling and recruitment	• Co-design recruitment strategies and materials with collaborators to ensure they are accessible and acceptable • Use the expertise of “on the ground” collaborators to target initial recruitment on “expert” individuals with experience of the phenomenon under study • Work with community organizations with established relations of trust with community members to facilitate rapid recruitment • Have contingency recruitment plans in place at the protocol stage to avoid the inevitable delays if plans are changed and new research governance amendments are needed
Data collection and analysis	• Focused research question should guide data collection and analysis • Team based, collaborative, iterative data collection and analysis • Conducting interviews online can facilitate rapid data collection but use alongside other methods to avoid exacerbating digital exclusion for some groups. • Pragmatic deductive and inductive data coding approaches are used to meet the aims of the study. • Interviews immediately analyzed at the end of the interview using a framework matrix (that covers study aims, main themes of interview topic guide, but also allows for inductive coding), from notes and listening to interview audio recording. • Initial analysis to focus on the needs of stakeholders, through concentrating on key critical issues and using these to write rapid reports. Subsequently, more in depth analysis from transcripts can guide academic publications. • Focus on a team-based approach to data collection and analysis, regular debrief between researchers, team reflexivity and data interpretation. • Where possible, follow the principles of co-production working with people with lived experience to co-create knowledge, helping to attribute meaning to findings and identifying key messages.
Dissemination of findings, and establishing impact	• When planning a project, allocate adequate time and resources to the dissemination phase • Early in the project, identify the key stakeholders who you want to influence with the research findings • Place the end users of research findings at the heart of the research implementation to ensure findings are appropriate and engaging for the intended audiences. • Two-stage approach to disseminating findings: 1. Rapid feedback loops to stakeholders and key audiences via meetings and rapid reports which focus on addressing stakeholders' questions/needs 2. Publication of journal articles directed toward the academic community and wider audiences. • Journal articles uploaded to preprint sites for immediate dissemination via social media • Work with stakeholders in relevant networks to disseminate research findings and create change.

### Project set up and management

To gain a rapid understanding of study context, CLIP-Q takes a collaborative approach, working with community partners, key stakeholders, and end-users to rapidly produce knowledge and generate findings grounded in practice. This has advantages for conducting research within tight deadlines by allowing the study team to rapidly familiarize themselves with the context of the study, identify stakeholders' perspectives on key questions to be explored during data collection and providing opportunities to engage and form links with participant groups early in the research process. Equally, potential barriers to recruitment can be identified and solutions found, and dissemination/impact strategies planned at the earliest stage. When possible, the CLIP-Q approach involves researchers, stakeholders, and members of the public with lived experience sharing responsibility and power from the start to the end of the projects to co-produce knowledge (Staniszewska et al., 2018).

Our Rapid COVID-19 Intelligence to Improve Primary Care Response (RAPCI) study was a longitudinal investigation into how GP practices were coping during the first COVID- 19 lockdown. The study had collaborators from Bristol, North Somerset and South Gloucestershire Clinical Commissioning Group (BNSSG CCG) and One Care (federation of 77 general practices). This enabled the researchers to understand the rapidly changing situation general practices faced at the start of the COVID-19 pandemic and the most pressing priorities for investigation.

Project set-up stage strategies can also help to overcome the potential challenges of multi-stakeholder collaborations. Collaborations within ARC West are formalized by a “Collaborative Project Outline” (CPO) document—an agreement between all parties that articulates the aims and scope of the project and the roles, responsibilities, and time commitment of individuals involved. The CPO ensures that stakeholders and researchers have shared expectations of the timeline and project goals. The early discussions required in order to formulate a CPO are important for identifying differences between partners and aligning priorities, e.g., clarifying needs for rapid service evaluation vs. the requirements for achieving academic rigor (Brewster et al., 2015).

Our Low Dead Space project examined whether people who inject drugs (PWID) were willing to switch to using a low dead space syringe to inject drugs. The study had collaborators from Bristol City Council, Public Health England, Bristol Drugs Project (BDP), a provider of harm reduction services, and PWID. They provided advice and guidance to rapidly familiarize the research team with the study setting. The CPO was important to clarify roles in order to form an equitable partnership at the start of the project, assisted by engaging in reflective practices during discussions to recognize and overcome any threats to equitable co-production.

### Establishing study aims

Traditionally qualitative research is exploratory and adopts a “wide-angle research lens” (Millen, 2000). By adopting close collaborations with stakeholders, a CLIP-Q study agrees a pragmatic focus on key research questions to inform rapid decision-making. Working closely with partners to flexibly adjust and narrow the focus of research questions is necessary to meet the needs of a rapidly changing context.

For our Low Dead Space Syringe project, BDP practitioners' and PWIDs' in-depth knowledge of current practices and service provision were used to inform the research aims in a timely and context-specific way. In the Back-to-School study, which examined the feasibility of school COVID-19 mitigation guidance, ARC West's established links to local education leaders, community groups and researchers with expertise in the field were vital in rapidly establishing focal study aims.

### Participant sampling and recruitment

Adopting a collaborative, focused lens to define research questions can also identify targeted purposeful and achievable sampling and recruitment strategies for rapid implementation. For our Low Dead Space Syringe project, working in collaboration with BDP was invaluable in co-designing feasible recruitment strategies. We found that providing study information in advance for an interview to be arranged at a later date did not fit well with PWID's lives, as it was difficult for them to predict when they would be available for interview. *Ad-hoc* opportunistic interviewing through intensive fieldwork was found to be more appropriate, with the researcher spending time on site conducting (up to five) interviews in succession. The assertive efforts and enthusiasm of BDP engagement workers facilitated this approach; practitioners explaining the study to service users and gaining their trust in the research team ensured participation from a diverse sample of PWID.

A targeted, purposeful approach to sampling and recruitment can enable a greater amount of useable data to be collected per participant, and thus fewer participants may be needed to address a specific research question (Millen, 2000). It has been suggested that the more “information power” the sample provides, the smaller the sample size needed (Malterud et al., 2016). Smaller samples can be sufficient if participants with experiences relevant to the research question are targeted.

Regular debrief meetings with collaborators are vital to take stock of recruitment and facilitate access to less-often-heard voices of individuals from marginalized groups. The Back-to-School project built multiple recruitment routes into the protocol to avoid delays waiting for amendments to research governance approvals. Our links with local education leaders meant our study recruitment pack was quickly sent to all schools in the region. We initially planned to recruit staff and families via schools, but many schools were unable to act swiftly due to competing demands of the pandemic. To recruit diverse families, the team worked with community groups linked to racially minoritised communities, who were more vulnerable to COVID-19 mortality and morbidity. These groups' established relations of trust with community members facilitated rapid participant recruitment. Using local and cultural expertise was pivotal in engaging with families and supporting them to understand the value of the research.

### Data collection and analysis

Traditional qualitative research can be criticized for taking a long time—too long for these urgent topics. To compress the time taken to collect and analyse data, CLIP-Q adopts an intensive team-based approach. ARC West has a large team of qualitative researchers working across multiple projects, who can temporarily be moved between studies to help focus on urgent priorities. Once the project team is assembled, good communication and regular meetings are vital to enable the team to rapidly undertake recruitment, discuss emergent findings and encourage team-based reflexivity (Rankl et al., 2021).

For the RAPCI study, a team of three qualitative researchers carried out concurrent interviews and analysis. During data collection an iterative approach with frequent feedback meetings between researchers allowed for exchange of experiences, discussion of findings, amendment of topic guides and modification of recruitment strategies. These meetings also encouraged the team to be reflexive, discussing from multiple perspectives any assumptions or interpretations that influenced conclusions drawn from the data. Due to the need to produce real time reports to the CCG, data analysis and write-up were conducted at speed, meaning there was insufficient time for interviews to be transcribed and transcripts fully coded. Accordingly rapid framework analysis was adopted, with a structured matrix produced to summarize key information and short illustrative quotations from the interviews. The team charted data directly into the framework matrix immediately following the interviews by listening to the audio recordings. In this way, a 30-min interview could be charted into the framework matrix within an hour of interview completion.

CLIP-Q analysis is driven by a pragmatic approach that relies on combining induction and deduction (Morgan, 2007; Skillman et al., 2019). The framework approach (Gale et al., 2013) was particularly suitable for the RAPCI study because interviews (although semi-structured) were highly focused rather than exploratory, prioritizing a small set of core topics and emerging issues. The study involved longitudinal interviews conducted in four fortnightly “rounds” and the focussed research questions evolved in each round depending on emerging findings and the changing priorities of NHS collaborators, driven by the evolving pandemic. The framework matrix was therefore a priori in the first round and tailored to the rapid reporting needs of the project in future rounds. Charting data into a framework matrix could proceed much more quickly than free coding transcripts but required researchers to balance the meaning and context of the data against the need to significantly condense and summarize the data effectively. Framework analysis also provided a structure to write up data.

For the Back-to-School, researchers worked closely with collaborators and used social media to keep informed of rapidly evolving COVID-19 policies and had to be agile during data collection—updating topic guides regularly—to ensure latest guidance changes were incorporated. Conducting interviews online, although a necessity due to COVID-19 restrictions, also facilitated rapid data collection as interviews could be arranged quickly, especially during lockdowns. However, there is a need to be cautious about who may be excluded if only using online interviews and at times the team paused recruitment to take stock, to ensure we had a range of perspectives. Interviews were immediately analyzed by the interviewer, from their own notes and the audio recordings. A framework matrix was used that covered the aims of the study and main themes of interview topic guide, but also facilitated for inductive coding. This rapid analysis was used to write a “living document” to produce rapid reports which facilitated early dissemination to key local and national stakeholders. Later when interview transcripts became available, the team added further details and direct quotes to the framework matrix to produce a journal article (Lorenc et al., 2021).

For the Low Dead Space Syringe project, BDP practitioners involved as co-producers used their expertise to inform the interview topics, ensuring key areas were covered and that the language employed resonated with interview participants. BDP's relationship with service users contributed to trust in the research team and thereby participants' willingness to discuss sensitive topics during interviews. Participation of PWID extended to co-creating knowledge with the researchers, helping in attributing meaning to findings and identifying key messages during analysis meetings; data are made meaningful in a collaborative process. This can help the research to be communicated to a wider audience thus maximizing impact.

### Dissemination of findings, and establishing impact

A crucial rationale for the CLIP-Q approach is being able to disseminate findings quickly to key audiences to inform decision making. We adopt a two-stage approach to writing and disseminating findings which involves: (1) rapid feedback loops to stakeholders and key audiences via meetings and rapid reports of emerging findings which focus on addressing stakeholders' questions/needs; (2) publication of journal articles, promoted via online news stories directed toward the academic community and wider audiences. As the review process for journals can take months, which can risk findings being less relevant when published (Baines and Gnanayutham, 2018), journal articles are uploaded to preprint sites for immediate dissemination via social media.

To enable early and continuous dissemination of findings, the RAPCI study rapid feedback loop to BNSSG CCG entailed presenting findings at weekly COVID-19 Primary Care Cell meetings, thereby informing the CCG's pandemic response and the future direction of the study. We also produced five rapid reports between May and July 2020, published online on the ARC West's website and disseminated to GP practices locally and nationally via local contacts, university communications channels and twitter. Using our collaborators' networks of contacts to disseminate findings and influence change, findings were included in reports to UK government's Scientific Advisory Group for Emergencies (SAGE), the Royal College of General Practitioners (RCGP) and NHS England. We also produced three academic papers from the study (Murphy et al., 2021; Scott et al., 2021; Turner et al., 2021).

For the Low Dead Space Syringe project, we worked with PWID to translate the research findings into co-designed accessible harm reduction material, refining the messages, language, and helping with dissemination and plans for implementation. Using the principles of co-production and placing PWID at the center of the process was essential to ensure the materials were appropriate, engaging, and did not stigmatize the intended audience. The co-designed process required a pragmatic and flexible approach by the team to ensure the disseminated materials met the end users' needs. The team also produced two academic papers outlining the main findings and the co-production process (Kesten et al., 2017; Hussey et al., 2019).

## Discussion

Key to CLIP-Q is a collaborative approach at all stages of the design, conduct and implementation of projects. Meaningful collaboration enables the diverse users of the findings to be active agents with equal standing to the researchers in designing, producing, and/or implementing research findings in a timely way (Heaton et al., 2016). Collaborations can focus the research questions on key real-world needs, take a purposeful and pragmatic approach to sampling and recruitment, and facilitate access to participants. Working closely with collaborators can also create a sense of ownership of the study findings, which can help dissemination and implementation (Vindrola-Padros, 2021). However, projects need to be properly resourced to enable meaningful stakeholder involvement, for example to assist with interpretation of findings and co-production of key messages. Resources are also needed to bring on board multiple experienced researchers with the skills required to share the workload of rapidly collecting and analyzing data in a robust manner (Taylor et al., 2018; Skillman et al., 2019; Vindrola-Padros and Johnson, 2020).

Another major feature of our CLIP-Q approach is intensive team-based data collection and analysis, with frequent team meetings and shared real-time data analysis through use of a joint analysis framework. The capacity for more than one researcher to work on the same study allows for faster data collection and analysis and enables peer quality control as well as exchange of expertise. Iterative rounds of data collection and the process of summarizing interview data directly from audio recordings immediately after interviews, allows team-based analysis to be conducted on a timescale that enables rapid feedback cycles to stakeholders to aid decision making (Mcnall and Foster-Fishman, 2007). Collaborative team-based working can improve analytic rigor when working at speed, with the process of examining data from multiple perspectives assisting collective interpretation of data, challenging assumptions about findings and encouraging team-based reflexivity (Beebe, 2001; Rankl et al., 2021). However, an open, trusting, flexible and non-hierarchical ethos is important to the success of team-based research to allow everyone to voice their opinions (Rankl et al., 2021). Producing rapid findings can generate extra internal and external pressures and be exhausting for the researchers involved, so supportive team working is essential to set realistic goals and share workloads (Vindrola-Padros and Johnson, 2020; Rankl et al., 2021).

Adopting a collaborative and intensive team-based approach to produce timely and relevant findings requires the research team to be pragmatic about what can be achieved with the time and resources available. This requires making compromises with collaborators to focus on key research questions, using flexible designs that can accommodate shifting needs and priorities and timely sharing of findings (Vindrola-Padros et al., 2021). This can produce tension between the quality standards of academic research and the demands and pressures placed by real world constraints. CLIP-Q pragmatic strategies to reduce data analysis timeframes include initial direct analysis from interview audio recordings and notes and team-based analysis to share workloads. Previous authors have compared similar techniques against conventional coding of full transcripts and found they identified the same broad themes, but with the added benefit of the rapid feedback loop with stakeholders allowing them to be a part of the analysis process (Burgess-Allen and Owen-Smith, 2010). However, this is reliant on having experienced researchers to conduct the rapid analysis and there may be the potential for not achieving the same “depth” or “level of interpretation” as conventional methods of data analysis (Vindrola-Padros and Johnson, 2020).

The CLIP-Q two-stage dissemination approach allows for both practical and academic interests to be met. First the needs of stakeholders and end users are met, through concentrating analysis on key critical issues and using these to write rapid reports and disseminating activities for a lay audience. Subsequently, more in depth analysis can ensue and guide later academic publications.

Qualitative applied public health and healthcare research is now taking place at a different pace and within a different paradigm from that of traditional academic research. There is a move from a scientific hegemony valuing academic knowledge, to embracing research collaboration and knowledge co-production by researchers working alongside stakeholders and service users to create findings that are rapid, responsive, and relevant (Riley et al., 2013; Vindrola-Padros et al., 2021). CLIP-Q uses a collaborative and intensive pragmatic team-based approach to focus research questions and guide strategies to enable efficient design and expedited data collection, analysis, and dissemination of urgent evidence to stakeholders as well as academic publications.

## Data availability statement

The original contributions presented in the study are included in the article/supplementary material, further inquiries can be directed to the corresponding authors.

## Ethics statement

The studies involving human participants were reviewed and approved by the Faculty of Health Sciences Committee for Research Ethics, University of Bristol (Low Dead Space Study, Ref 19861), (Back-to-School study, Ref 108084), (The RAPCI study, Ref 103166). The RAPCI study was also approved by the Health Research Authority approval: IRAS project ID: 282541, REC reference: 20/HRA/2070. Written informed consent to participate in this study was provided by the participants' legal guardian/next of kin.

## Author contributions

All authors listed have made a substantial, direct, and intellectual contribution to the work and approved it for publication.

## Funding

This research was funded by the National Institute for Health and Care Research Applied Research Collaboration West (NIHR ARC West).

## Conflict of interest

The authors declare that the research was conducted in the absence of any commercial or financial relationships that could be construed as a potential conflict of interest.

## Publisher's note

All claims expressed in this article are solely those of the authors and do not necessarily represent those of their affiliated organizations, or those of the publisher, the editors and the reviewers. Any product that may be evaluated in this article, or claim that may be made by its manufacturer, is not guaranteed or endorsed by the publisher.

## Author disclaimer

The views expressed in this article are those of the authors and not necessarily those of the NIHR, or the Department of Health and Social Care.
